# Assessment of Met and Unmet Care Needs in Older Adults without Mental Disorders Using the Camberwell Assessment of Need for the Elderly: A Systematic Review and Meta-analysis

**DOI:** 10.34172/jrhs.2021.64

**Published:** 2021-10-12

**Authors:** Parvin Cheraghi, Ahmad Delbari, Zahra Cheraghi, Akram Karimi-Shahanjarini, Nasibeh Zanjari

**Affiliations:** ^1^Department of Gerontology, University of Social Welfare and Rehabilitation Sciences, Tehran, Iran; ^2^Iranian Research Centre on Aging, University of Social Welfare and Rehabilitation Sciences, Tehran, Iran; ^3^Department of Epidemiology, School of Public Health, Hamadan University of Medical Sciences, Hamadan, Iran; ^4^Modeling of Noncommunicable Diseases Research Center, Hamadan University of Medical Sciences, Hamadan, Iran; ^5^Department of Public Health, Social Determinants of Health Research Center, Hamadan University of Medical Sciences, Hamadan, Iran

**Keywords:** Aged, Meta-Analysis, Needs Assessment, Systematic Review

## Abstract

**Background:** Physical, psychological, and social changes in the aging lead to new needs in the care of the elderly. The Camberwell Assessment of Need for the Elderly (CANE) evaluates older adults' care needs. This study aimed to assess the types of needs of the elderly using the CANE questionnaire.

**Study design:** A systematic review.

**Methods:** This systematic review included all cross-sectional studies. International databases, including Web of Sciences, Medline, Scopus, and ProQuest were searched up to June 2021. Such keywords as aged OR ageing OR "older adults" OR "older people" OR "older person" OR elderly, AND need OR "needs assessment" OR "met needs" OR "unmet needs" were used to design the search strategy. A 95% CI was calculated using the exact method, and the meta-analysis of proportion (metaprob) module was used for data analysis.

**Results:** In total, 769 studies were retrieved in this review. At the following stages, 760 articles were excluded upon checking the duplicates; moreover, the titles and abstracts did not meet the eligibility criteria. Finally, nine studies remained. The mean±SD age of 2200 participants was obtained at 78.4±5.9 years. The highest and lowest met needs were related to the physical (45%) and social (21%) dimensions, respectively. Furthermore, the highest unmet needs were observed in the physical and social dimensions (0.07%), and the lowest unmet needs were related to the psychological and environmental dimensions (0.04%).

**Conclusions:** The CANE is sensitive enough to identify unmet needs in different samples and settings. Therefore, a new care model and appropriate interventions for the elderly can be designed based on the CANE results.

## Introduction

The increase in the elderly population is a global phenomenon and a fundamental challenge for the health system of any community^1,2^. In addition to physical disorders, the elderly face psychological, social, environmental, and care problems; moreover, they often have complex and unknown needs ^3^. On the other hand, issues, such as lack of security, lack of social participation, inadequate living environment, and psychological problems have been mentioned as significant needs ^4^.

The needs assessment should be comprehensive, multidimensional, and systematic to ensure that individuals' unmet needs are well identified ^1^. The issue of meeting the unique needs of the elderly in the community to promote active and successful aging is gaining new and broader dimensions every day ^3^. Identification of the real needs of the elderly and development of plans to provide them better and more practically can be considered one of the actions of service centers for the elderly. A comprehensive needs assessment can also help focus on the health care workforce on the area of identified needs^5^. Finally, identifying needs increases the quality of life and the sense of satisfaction of the elderly. It also prevents the elderly from staying in long-term care centers and hospitalization and reduces their mortality rate ^6^. The experience of the developed countries has shown that the unmet needs of the elderly can place a heavy burden on social, economic, and health systems ^7^. Therefore, countries facing a wave of aging in the future should identify the unknown needs and take appropriate measures to meet their needs ^8^. The Camberwell Assessment of Need for the Elderly (CANE) questionnaire has 24 items in the area of needs related to the elderly and covers met and unmet needs in the areas of social, psychological, and physical health, as well as environmental needs among elderly. This questionnaire was first developed for use in the elderly with mental disorders and then used for elderly without mental disorder. Reynolds et al. first developed the CANE questionnaire in 2000 based on the Camberwell Assessment of Need questionnaire to assess the needs of adults with mental health disorders ^5^. Hancock and Orrell released their last revision in 2004^9^. The validity and reliability of this questionnaire have been examined considering the elderly population of several countries, and there is general agreement that CANE covers the main dimensions of the needs of older adults^5,6,10,11^. One of the main advantages of CANE is a comprehensive approach to the needs of the elderly in economic, social, psychological, and physical dimensions, and notices the need as a lack of service. Furthermore, an essential feature of this tool is identifying unmet needs for which appropriate interventions have not yet been made^5,6,12-16^. Accordingly, this study was conducted to assess the met and unmet needs of the elderly using the CANE questionnaire in a structured (systematic) review study.

## Methods

###  Eligibility criteria

 In this systematic review, all cross-sectional studies were included to estimate the overall prevalence of met and unmet needs in the elderly. The study population in this review was the elderly population of the world regardless of gender and ethnicity. There were no restrictions on the time, location, and language of the studies.

###  Search Strategy

 Such keywords as aged OR ageing OR "older adults" OR "older people" OR "older person" OR elderly, AND need OR "needs assessment" OR "met needs" OR "unmet needs" were used to design the search strategy. National and international databases, as well as Web of Sciences, Medline, Scopus, and ProQuest were searched up to June 2021.

The reference lists of all retrieved studies were scanned in order to find additional references (e.g., https://ec.europa.eu/eip/ageing/events/16th-international-conference-integrated-care-movement-change-enabling-people-centred-and_en.html).Two investigators (P. Ch and Z. Ch) were independently and simultaneously responsible for screening the titles and abstracts of the retrieved studies. In case of any disagreement, it was resolved upon discussion and judgment of a third investigator (A.D).

 In addition, the Kappa index was calculated in order to evaluate the agreement rate of the investigators. The inter-authors reliability based on Kappa statistics was obtained at 85%. Afterward, the full texts of the selected studies were reviewed to assess the eligibility criteria. Finally, the studies that met the inclusion criteria were selected for analysis.

###  Data extraction

 Two authors (P.Ch and Z.Ch) extracted the data from the included studies. The following data were extracted using a pre-designed datasheet from the studies that met the inclusion criteria (the first author’s name, year of publication, location of study, mean age of the participant, gender, and sample size). In case of missing data in the included studies, the authors were contacted.

###  Risk of bias assessment

The quality of the included studies was assessed using the CASP checklist ^17^. The following items were used for quality assessment: (1) presence of a focused question; (2) appropriateness of the method for answering the research question; (3) description of methods of selection; (4) un-biases of sampling; (5) representative of sampling; (6) sample size based on pre-study considerations of statistical power; (7) satisfactory response rate; (8) measurements likely to be valid and reliable; (9) assessment of the statistical significance; (10) confidence intervals given for the main results; (11) confounding factors that have not been accounted for; and (12) applicability of the results to the organization. The score range of the questionnaire was from 0 to 24 (0-12, 13-18, and above 19 were rated as poor, moderate, and good qualities, respectively).

###  Assessment of heterogeneity

 The statistical heterogeneity was checked using the Chi-square test at a 10% significance level. Moreover, the heterogeneity was quantified using the I2 statistics. The between-study variance was estimated using tau-square (Ta2). The extracted data recheck, meta-regression, and subgroup analysis were the utilized approaches to deal with the heterogeneity.

###  Data analysis

 The percent of met and unmet needs was used in each study as the main statistic. In some studies that did not report the percent, it was calculated by dividing the number of participants by the sample size. The standard error of prevalence was calculated as follows:


$$\sqrt{\frac{p\times (1-p)}{n}}$$

The inverse variance method was used to obtain the pooled prevalence. In the studies in which prevalence was close to zero or one, 95% CI was calculated using the exact method, and the meta-analysis of proportion (metaprop) module was utilized for data analysis. The random-effects model was employed for reporting the results at 95% CI. The Stata 11 (Stata Corp, College Station, TX, USA) was used for data analysis.

## Results

 In this review, 769 studies were retrieved through international databases, and 412 were excluded because of duplication. In the next stage, 226 articles were excluded upon checking the titles and abstracts, and another 122 studies were excluded upon checking the full texts as they did not meet the eligibility criteria.

**Figure 1:**
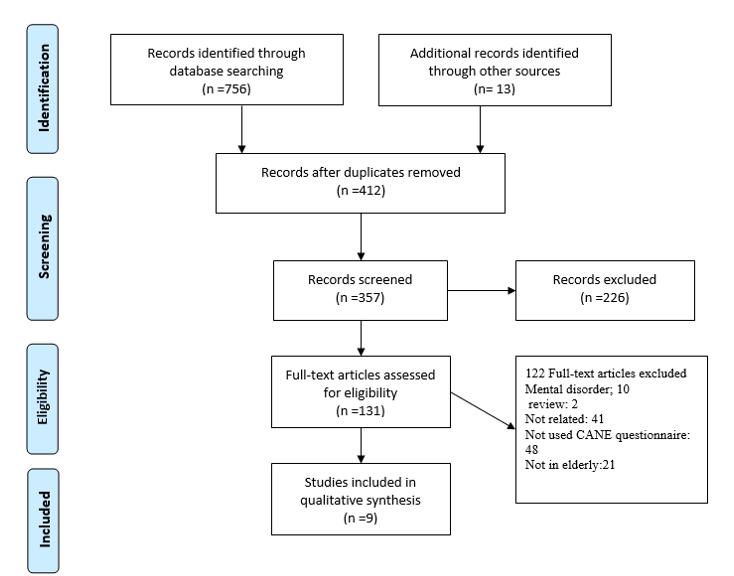
Identification of the included studies

Finally, nine studies ^1,14-16,18-22^ remained in the final analysis (Figure 1). The total sample size was 2200 subjects. The mean ±SD age of the participants was obtained at 78.4 ±5.9 years (Table 1).

**Table 1:** Characteristics of the included studies

**Authors**	**Year**	**Country**	**Samplesize**	**Gender**	**Age range(yr)**	**Mean age**	**Quality**
Gholizadeh	2020	Iran	240	Both	≥60	68.1	High
Sousa	2008	Brazil	32	Both	65 to 88	72.8	Moderate
Stein	2013	Germany	158	Both	68 to 98	80.3	High
Stein	2016	Germany	816	Both	75 to 98	80.4	High
Tiativiriy	2017	Thailand	330	Both	60 to 100	70.3	High
Tobis	2016	Poland	173	Both	75 to 102	82.7	Moderate
Wieczorowska-Tobis	2018	Poland	306	Both	75 to 108	83.2	High
Ploeg	2013	The Netherlands	93	Both	72 to 98	86.7	High
Walters	2000	England	52	Both	75 to 95	81.5	Moderate

###  Heterogeneity

 I2 and Chi-square (at a significance level of 0.05) tests were used for quantitative and qualitative heterogeneity, respectively. Moreover, the tau-squared test was used to estimate the variances among the studies. In all the analysis subgroups (met and unmet needs), considerable heterogeneity (over 90%) was observed. These inconsistencies were also found to be significant with the Cochrane test (P<0.001). These results have been observed in the cross-sectional and cohort studies (graphs 1-4).

###  Data gathering and validity assessment of studies

 The risk of bias (quality) of the included publications was also appraised using the CASP checklist. The same investigators (P.Ch. and Z.ch) appraised the studies independently. Based on the recommended items of the CASP checklist, the cross-sectional studies of high quality (66.70%) and intermediate quality (33.3%) were classified.

###  Estimated pooled proportion of met and unmet needs

**Figure 2:**
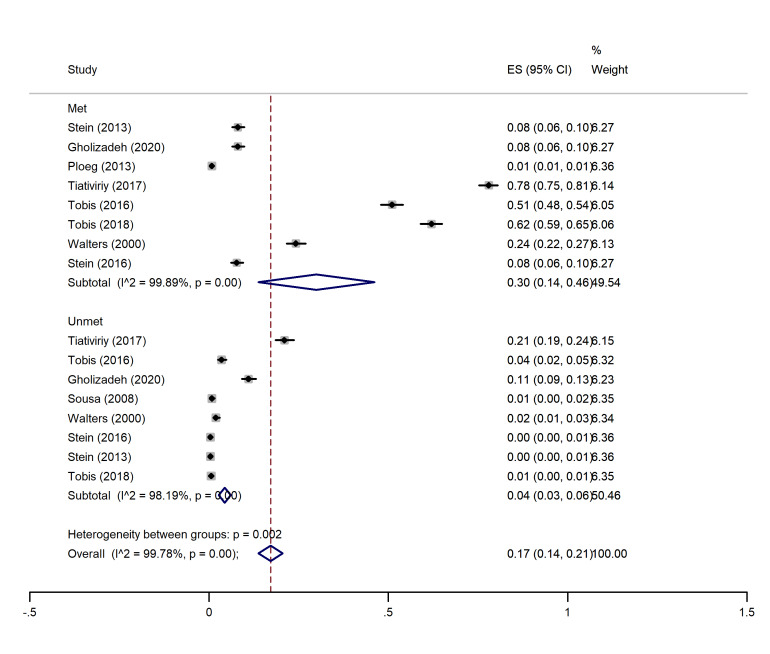
Forest plot of the percent of met and unmet environmental needs in elderly

**Figure 3:**
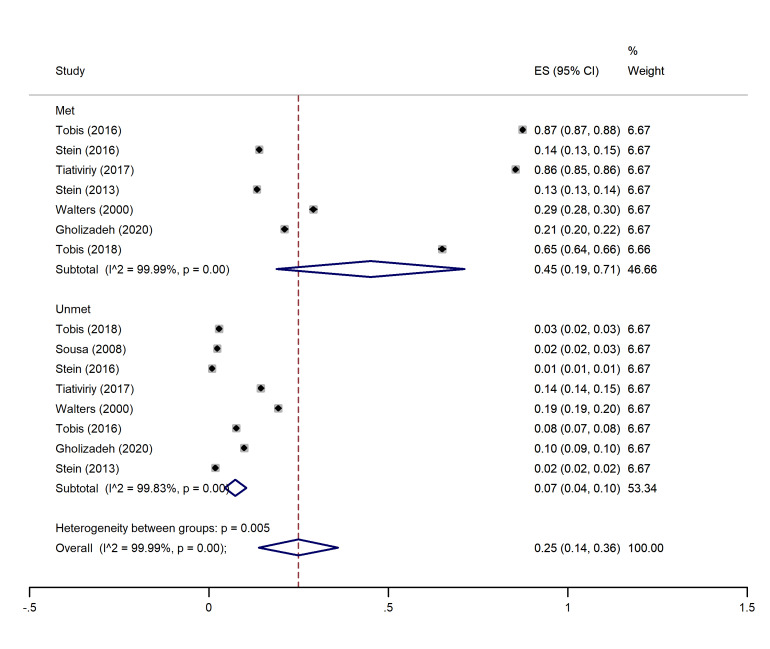
Forest plot of the percent of met and unmet physical needs in elderly

**Figure 4:**
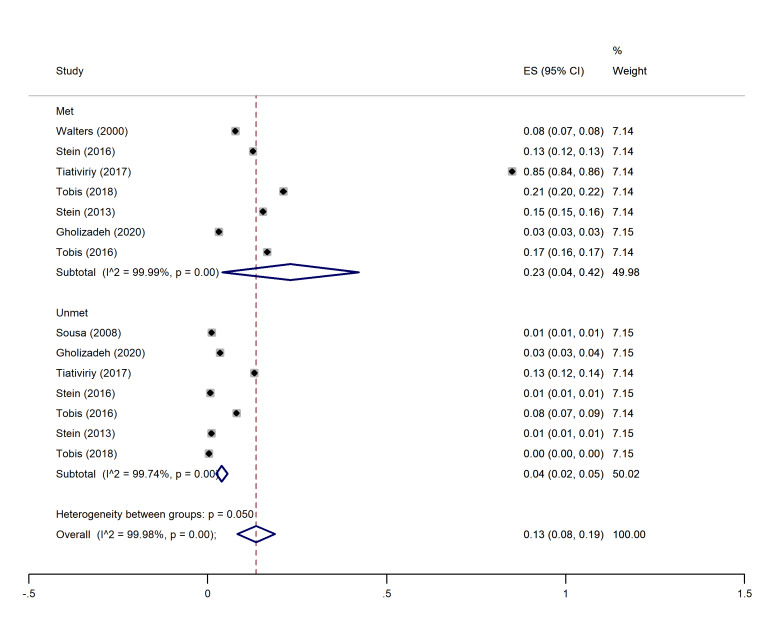
Forest plot of the percent of met and unmet psychological needs in elderly

**Figure 5:**
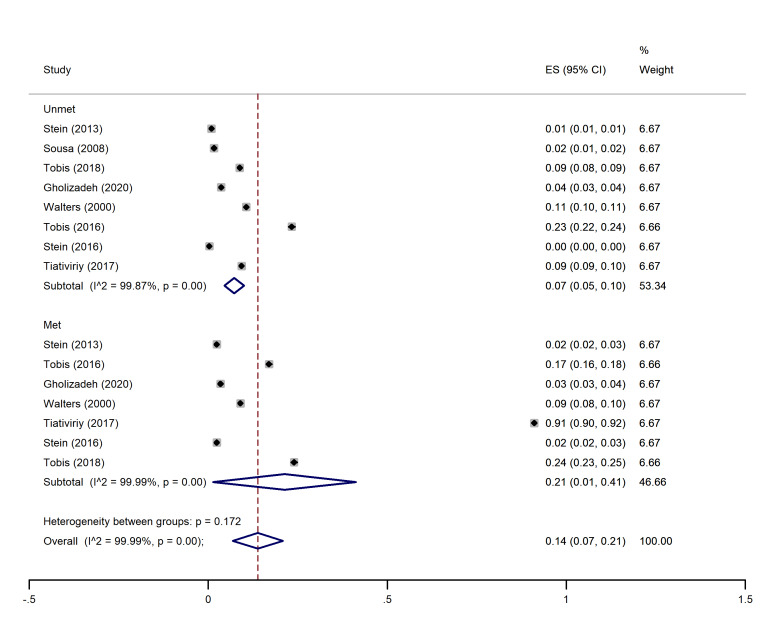
Forest plot of the percent of met and unmet social needs in elderly

The highest and lowest met needs were related to the physical (45%, 95% CI: 0.19-0.71) and social (21%, 95% CI: 0-01.41) dimensions, respectively. Moreover, the highest unmet needs were observed in the physical (7%, 95% CI: 0.04-0.10) and social dimensions (7%, 95% CI: 0.05-0.10), and the lowest unmet needs were related to the psychological (04%, 95% CI: 0.02- 0.05) and environmental dimensions (04%, 95% CI: 0.03- 0.06) (See Figures 2-5).

###  Pooled proportion of environmental needs

In this dimension, nine studies ^1,14-16,18-22^ had indicated the percentage of met and unmet needs of the environmental dimension. The overall pooled proportion of environmental met needs was 30% (95% CI: 0.14-0.46), and the overall pooled proportion of unmet environmental needs was 0.04 (0.03, 0.06) (Figure 2).

###  Pooled proportion of physical needs

In this dimension, eight studies ^1,14-16,18,20-22^ had indicated the percentage of met and unmet needs of the physical dimension. The overall pooled proportion of physical met needs was 45% (95% CI: 0.19-0.71), and the overall pooled proportion of unmet physical needs was 0.07 (0.04, 0.10) (Figure 3).

###  Pooled proportion of psychological needs

In this dimension, eight studies ^1,14-16,18,20-22^ had indicated the percentage of met and unmet needs of the psychological dimension. The overall pooled proportion of psychological met needs was 23% (95% CI: 0.04-0.42), and the overall pooled proportion of unmet psychological needs was 0.04 (0.02, 0.05) (Figure 4).

###  Pooled proportion of social needs

In this dimension, eight studies ^1,14-16,18,20-22^ indicated the percentage of met and unmet social dimension needs. The overall pooled proportion of social met needs was 21% (95% CI: 0.01-0.41), and the overall pooled proportion of unmet social needs was 0.07 (0.05, 0.10) (Figure 4).

## Discussion

This study was conducted to assess the needs of the elderly using the CANE questionnaire. The estimated met and unmet needs of the elderly after a review study led to a meta-analysis in four areas of environmental or basic needs, physical, psychological, and social. After analyzing the final studies, the highest and lowest met needs were in the physical (43%) and environmental dimensions (21%), respectively. The high estimated physical needs may be due to the inclusion of older adults over 75 years of age who were cared for in Long Term Cares (LTC) in most of the final studies, and these settings appear to be institutions that take adequate care of service providers. In addition, this residential care is designed to meet the needs of their residents ^23,24^.

Regarding the low environmental needs, studies show that the environment and living conditions play a significant role in the estimated needs of the elderly. Therefore, the needs of the elderly who were residents in LTC settings and nursing homes, and hospitalized may be fundamentally different from the needs of the elderly living in the community ^9,23^. It is crucial to address basic needs in geriatric care interventions as unmet basic needs, such as safety, housing, and finances are strong predictors of depression^25^. Hospitalization and separation from home are also significant risk factors for depression in the elderly ^14,26,27^.

Among the unmet needs, the highest need was related to physical and social needs (0.06%), and the lowest unmet needs were related to the psychological dimension (0.03%). The results of some studies showed that the highest percentage of unmet physical needs was related to eyesight/hearing impairment, mobility/fall, and incontinence. Additionally, in social needs, most needs were related to company, intimate relationships, and daytime activities ^1,15,20^. It seems that the changes that occur in the physical dimension of the elderly cause limitations and adverse effects on their social activities. Since most of the elderly in this study lived in care centers, separation from their place of residence and relatives and unmet physical needs could be one of the reasons for their high unmet social needs.

Other results of this study included the relationship among unmet needs and depression, male gender, long-term care, relative care, medications, as well as mental and functional disorders ^1,14,19^. High dependency on elderly care is a strong predictor of increased unmet needs. There was also a correlation between psychological symptoms and needs. Symptoms that need treatment increase the need, and if these needs remain unmet, they can increase the symptoms of mental disorders ^14^.

One of the reasons for the low unmet needs in the field of psychology in the present study is the participants, which included the elderly who did not have problems with mental disorders. Moreover, in some studies, people with depression and severe cognitive disorders were excluded from the study using the Geriatric Depression Scale and the Mini-Mental State Examination^1^.

 Regarding the limitations of the study, it can be stated that the CANE questionnaire investigates the elderly, as well as formal and informal caregivers to assess the need. However, in the present study, due to the lack of reporting the views of formal and informal caregivers in all studies, the results of this review were reported only from the perspective of the elderly, which may be due to their various problems (cognitive and physical disorders), and high or low estimated needs. The difference between unmet needs from informal caregivers and the elderly depend on their perception of the needs. Therefore, a close estimate will be obtained when the opinions of both groups (elderly, as well as formal and informal caregivers) are reported and compared together. Another limitation is the low number of studies conducted with the CANE questionnaire and the lack of studies from around the world with different socio-economic statuses. Needs assessment results may also differ due to differences in the level of education of the elderly in developing and developed countries.

 Since the priority and number of the needs may be different from the type of participants and the type of setting, it is suggested that further studies be conducted on different age groups and elder adults with different degrees of physical and mental status, as well as in different settings and communities. It should also be performed better to identify the priority and unmet needs of the elderly and use their results in interventions and decisions to health promotion of the elderly. Finally, the publication bias was not estimated in this study since the nature assessing the likelihood of publication bias in the meta-analysis of prevalence studies is controversial.

## Conclusion

 Along with global changes in economic, social, and technological dimensions, the living environment and the physical and mental status of the elderly are also undergoing numerous changes. These changes have led to the emergence of unknown or unmet needs in various areas of care. Identifying and being responsible for addressing these needs is one of the main tasks of geriatric health policymakers and their formal/informal caregivers. CANE is a tool that can help identify the various dimensions of the needs of the elderly in any environment. Moreover, the use of the obtained results with the help of primary health care lead to the utilization of a new care model that can be presented based on appropriate interventions in the medical and social fields of the elderly.

## Acknowledgments

 The authors would like to thank the Research and Technology Deputy of University of Social Welfare and Rehabilitation Sciences, Tehran, Iran, for financial support (Code: 951196002, IR. USWR. REC.1398.126),

## Conflict of interests

 The authors declare that they have no conflict of interest regarding the publication of the current article.

## Funding

 This study was funded by the Research and Technology Deputy of University of Social Welfare and Rehabilitation Sciences, Tehran, Iran. The funders had no role in study design, data collection and analysis, decision to publish, or preparation of the manuscript.

 Highlights

- Physical and social dimensions are the highest and lowest needs met in elderly, respectively.
- Physical and psychological dimensions are the highest and lowest unmet needs, respectively.
- The CANE is sensitive enough to identify the needs of the elderly in each setting.
